# Three Related Enzymes in Candida albicans Achieve Arginine- and Agmatine-Dependent Metabolism That Is Essential for Growth and Fungal Virulence

**DOI:** 10.1128/mBio.01845-20

**Published:** 2020-08-11

**Authors:** Katja Schaefer, Jeanette Wagener, Ryan M. Ames, Stella Christou, Donna M. MacCallum, Steven Bates, Neil A. R. Gow

**Affiliations:** a Medical Research Council Centre for Medical Mycology at The University of Exeter, University of Exeter, Exeter, United Kingdom; b The Aberdeen Fungal Group, Institute of Medical Sciences, University of Aberdeen, Aberdeen, United Kingdom; c Department of Biomedical Sciences, Faculty of Science, University of Sheffield, Sheffield, United Kingdom; d Department of Infection, Immunity and Cardiovascular Disease, Medical School, University of Sheffield, Sheffield, United Kingdom; e Bateson Centre, University of Sheffield, Sheffield, United Kingdom; f Assay Development Scientist, Osler Diagnostics Limited, Oxford, United Kingdom; g Department of Biosciences, University of Exeter, Exeter, United Kingdom; Geisel School of Medicine at Dartmouth

**Keywords:** *Candida*, arginase, guanidinobutyrase, agmatinase, immunity, morphogenesis, *Candida albicans*, macrophages

## Abstract

Amino acid metabolism is crucial for fungal growth and development. Ureohydrolases produce amines when acting on l-arginine, agmatine, and guanidinobutyrate (GB), and these enzymes generate ornithine (by arginase), putrescine (by agmatinase), or GABA (by 4-guanidinobutyrase or GBase). Candida albicans can metabolize and grow on arginine, agmatine, or guanidinobutyrate as the sole nitrogen source. Three related C. albicans genes whose sequences suggested that they were putative arginase or arginase-like genes were examined for their role in these metabolic pathways. Of these, *Car1* encoded the only bona fide arginase, whereas we provide evidence that the other two open reading frames, orf19.5862 and orf19.3418, encode agmatinase and guanidinobutyrase (Gbase), respectively. Analysis of strains with single and multiple mutations suggested the presence of arginase-dependent and arginase-independent routes for polyamine production. *CAR1* played a role in hyphal morphogenesis in response to arginine, and the virulence of a triple mutant was reduced in both Galleria mellonella and Mus musculus infection models. In the bloodstream, arginine is an essential amino acid that is required by phagocytes to synthesize nitric oxide (NO). However, none of the single or multiple mutants affected host NO production, suggesting that they did not influence the oxidative burst of phagocytes.

## Abstract (precis)

We show that the C. albicans ureohydrolases arginase (Car1), agmatinase (Agt1), and guanidinobutyrase (Gbu1) can orchestrate an arginase-independent route for polyamine production and that this is important for C. albicans growth and survival in microenvironments of the mammalian host.

## INTRODUCTION

**FIG 1:**
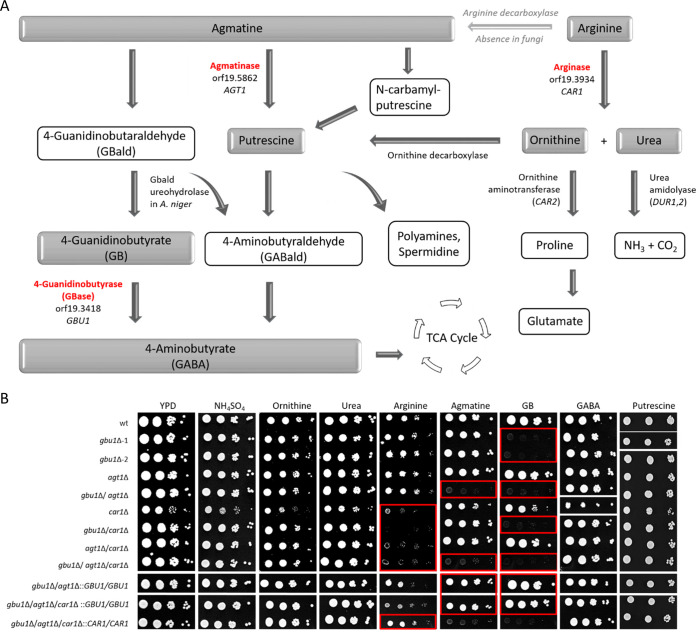
Arginase and agmatine metabolism mediated by the members of the C. albicans arginase-like gene family. (A) Simplified schematic pathway showing the catabolic steps and products of agmatine and arginine metabolism. TCA, tricarboxylic acid. (B) Spot assay of C. albicans ureohydrolase mutants on media containing intermediate products of the pathway as the sole nitrogen source. The inoculum and serial 10-fold dilutions of the C. albicans wild type plus single, double, and triple mutants of orf19.3934 (*CAR1*), orf19.5862 (*AGT1*), and orf19.3418 (*GBU1*) were spotted onto agar plates, incubated at 30°C, and imaged after 72 h (with the exception of YPD and arginine plates, which were imaged at 48 h and 96 h, respectively). Mutant phenotypes identified were confirmed subsequently by screening the complemented strains. Sections highlighted with a red rectangle identify reduced growth of the mutants. Representative images of colony growth are displayed, with white lines indicating the locations where the images were taken (i.e., from different plates or from different regions of the same plate), and were repositioned for presentation purposes.

l-Arginine is a conditionally essential amino acid in humans that plays important roles in cell division, wound healing, removal of ammonia from the body, immune function, and the release of hormones (1). It is a precursor for the synthesis of nitric oxide, which is an important antimicrobial component of the oxidative burst of immune phagocytes (2) and is important for the regulation of blood pressure and circulation by promoting blood vessel relaxation (3). Arginine occurs universally across all kingdoms of life and plays a key role in host-microbe interactions. In fungi, the arginase enzyme contributes to the major catabolic path for l-arginine (4, 5) whereas higher eukaryotes express arginine decarboxylase (6), which participates in an alternative (arginase-independent) pathway for arginine catabolism. This branch of arginine metabolism produces agmatine, the decarboxylated form of arginine, which is converted to putrescine and urea by the enzyme agmatinase (Fig. 1A). With some exceptions, exemplified by Ceratocystis minor, Verticillium dahliae (7), Gigaspora rosea (8), and Panus tigrinus (9), most fungi apparently do not have an arginine decarboxylase. Instead, the majority of fungi produce putrescine via the decarboxylation of ornithine, a product of the arginase reaction.

Very little is known about agmatine catabolism in fungi. Agmatinases belong to the family of ureohydrolases and generate urea as a common product. This enzyme family is characterized by their activity in catalyzing hydrolysis of the amide bond of l-arginine, agmatine, and guanidinobutyrate (GB) via arginase, agmatinase, and 4-guanidinobutyrase (GBase), respectively. Mostly characterized in bacteria (10, 11), agmatinases are metalloenzymes which hydrolyze agmatine to putrescine and are crucial in the alternative pathway used to produce polyamines in addition to the constitutive pathway using ornithine decarboxylase (ODC). Putrescine is then used to form polyamines such as spermine or spermidine (12) or is converted to 4-aminobutyrate (GABA) via 4-aminobutyraldehyde (GABald). In humans, agmatine is a cell signaling molecule that is involved in a number of cellular processes, including the innate immune response (13–17). Unlike in bacteria or humans, the functional importance of agmatine and its metabolism in fungi is not well understood.

Agmatine is present in the lumen of the human gastrointestinal tract and is derived from bacteria of the gut microflora (18), ingested food (19), or desquamated intestinal epithelial cells (18). The opportunist pathogen Candida albicans is a normal part of the gut microflora (20) but can also cause systemic candidiasis in immunocompromised patients or patients that have experienced trauma and medical interventions (21). C. albicans is able to survive and grow in a variety of microenvironments in the human host that differ substantially in nutrient composition (22).

The rapid increase in the use of whole-genome sequencing has enabled identification of putative arginase-like enzymes in many eukaryotes. However, gene identification of these enzymes, based exclusively on sequence homology, does not enable definitive predictions to be made with respect to the catalytic function of the encoded proteins. The C. albicans genome contains an annotated gene (orf19.3934) encoding a cytosolic arginase, *CAR1* (EC 3.5.3.1), and two uncharacterized open reading frames (ORFs), orf19.3418 and orf19.5862, which were predicted to encode secreted proteins that display similarity to arginases (see Fig. S1A and B in the supplemental material). In order to identify the role of these genes in C. albicans arginine and agmatine metabolism, we generated single, double, and triple mutants of the corresponding ORFs using two independent knockout strategies, including the mini-URA blaster gene knockout approach (23) and the CRISPR-Cas9 technology for gene disruption or editing (24). We identify *CAR1* as the only bona fide arginase gene in C. albicans, whereas we provide evidence that the other two open reading frames, orf19.5862 and orf19.3418, likely encode agmatinase (Agt1) and guanidinobutyrase (Gbu1), respectively. This arginase-like gene family therefore contributes a set of ureohydrolases required for arginine and agmatine utilization. We demonstrate that this gene family is important for virulence in two independent animal infection models. Together, they play a key role in enabling C. albicans growth and survival in microenvironments of the mammalian host.

10.1128/mBio.01845-20.1 FIG S1 The C. albicans genome reveals two ORFs (orf19.5862 and orf19.3418) that show similarity to the annotated arginase gene *CAR1*. Data represent protein alignment of sequences (Agt1 and Gbu1) found via BLASTp analysis of C. albicans Car1 (A) and percentages of protein similarity (B), showing residues that are fully conserved (*), conservation between groups of strongly similar properties (:), or conservation between groups of weakly similar properties (.). The images were created by the use of Clustal2.1. Comparative genomics analyses resulted in a phylogenetic tree constructed using fungal 18S species from Yarza et al. (P. Yarza, P. Yilmaz, K. Panzer, F. O. Glöckner, and M. Reich, Mar Genomics 36:33–39, 2017, https://doi.org/10.1016/j.margen.2017.05.009) (C) that shows the presence (green) or absence (red) of orthologues in fungal species. Three species show an absence (loss or lack of a gain) of the two newly identified ORFs. P. jirovecii lacks orthologues of all these genes. For gene identifier (ID), length, E value, and bit score data for the orthologues, see Table S1. Download FIG S1, TIF file, 0.2 MB. Copyright © 2020 Schaefer et al. 2020 Schaefer et al. This content is distributed under the terms of the Creative Commons Attribution 4.0 International license.

## RESULTS

### C. albicans orf19.5862 and orf19.3418 function in agmatine catabolism.

We investigated the role of three gene products in Candida albicans, one that is annotated as an arginase (Car1) and two products of ORFs (orf19.5862 and orf19.3418) with similarity to arginase enzymes (see Fig. S1A and B in the supplemental material) but that had not been functionally annotated. Comparative genomics (Fig. S1C) revealed orthologues of all three ORFs in Candida dubliniensis, Candida tropicalis, Candida parapsilosis, Candida auris, Talaromyces marneffei, Aspergillus fumigatus, and Cryptococcus neoformans (see Table S1 in the supplemental material). An arginase orthologue was present in Saccharomyces cerevisiae, Candida glabrata, *and* Histoplasma capsulatum, but no equivalent of either of the two newly identified ORFs, orf19.3418 and orf19.5862, was identified. All three ORFs were absent in the obligate parasite Pneumocystis jirovecii. In order to facilitate identification of the role of these arginase-like genes in C. albicans, we generated single, double, and triple mutants with mutations of the corresponding ORFs using the CRISPR-Cas9 gene editing technology (24) and the mini-URA blaster gene deletion method (23). Mutants created in this work are listed in Table 1 and were ultimately named the *gbu1*Δ, *agt1*Δ, and *car1*Δ mutant strains, corresponding to mutants with mutations in ORFs orf19.3418, orf19.5862, and orf19.3934, respectively. Single mutants were created for each of the ORFs, and these were then used to create double and triple mutants. In addition, these mutants were complemented by reintroduction of the required wild-type genes into the null mutant background.

**TABLE 1:** C. albicans strains used in this study^*a*^

Strain	Genotype	Source
SC5314	wt; clinical blood isolate	Gillum et al. (63)
*gbu1*Δ*-1* NGY700	As SC5314 but *gbu1*Δ *gbu1*Δ	This work
*gbu1*Δ*-2* NGY701	As SC5314 but *gbu1*Δ *gbu1*Δ	This work
*agt1*Δ NGY702	As SC5314 but *agt1*Δ *agt1*Δ	This work
*gbu1*Δ/*agt1*Δ NGY703	As SC5314 but *gbu1*Δ *gbu1*Δ *agt1*Δ *agt1*Δ	This work
*car1*Δ NGY704	As SC5314 but *car1*Δ *car1*Δ	This work
*car1*Δ/*gbu1*Δ NGY705	As SC5314 but *car1*Δ *car1*Δ *gbu1*Δ *gbu1*Δ	This work
*car1*Δ/*agt1*Δ NGY706	As SC5314 but *car1*Δ *car1*Δ *agt1*Δ *agt1*Δ	This work
*gbu1*Δ/*agt1*Δ/*car1*Δ NGY707	As SC5314 but *car1*Δ *car1*Δ *gbu1*Δ *gbu1*Δ *agt1*Δ *agt1*Δ	This work
*gbu1*Δ/*agt1*Δ+*gbu1* NGY708	*gbu1*Δ *gbu1*Δ *agt1*Δ *agt1*Δ:: *GBU1 GBU1*	This work
*gbu1*Δ/*agt1*Δ/*car1*Δ+*gbu1* NGY709	*gbu1*Δ *agt1*Δ *car1*Δ:: *GBU1 GBU1*	This work
*gbu1*Δ/*agt1*Δ/*car1*Δ+*car1* NGY710	*gbu1*Δ *agt1*Δ *car1*Δ:: *CAR1 CAR1*	This work
*gbu1*Δ SBC199	As CAI4 but *gbu1*Δ::*dpl200 gbu1*Δ::*dpl200 RPS1 rps1*Δ::CIp10	This work
*agt1*Δ SBC200	As CAI4 but *agt1*Δ::*dpl200 agt1*Δ::*dpl200 RPS1 rps1*Δ::CIp10	This work
*car1*Δ SBC201	As CAI4 but *car1*Δ::*dpl200 car1*Δ::*dpl200 RPS1 rps1*Δ::CIp10	This work
*gbu1*Δ/*agt1*Δ SBC202	As CAI4 but *gbu1*Δ::*dpl200 gbu1*Δ::*dpl200 agt1*Δ::*dpl200 agt1*Δ::*dpl200 RPS1 rps1*Δ::CIp10	This work
*gbu1*Δ/*agt1*Δ/*car1*Δ SBC203	As CAI4 but *gbu1*Δ::*dpl200 gbu1*Δ::*dpl200 agt1*Δ::*dpl200 agt1*Δ::*dpl200 car1*Δ::*dpl200 car1*Δ::*dpl200 RPS1 rps1*Δ::CIp10	This work
*gbu1*Δ+*GBU1* SBC204	As CAI4 but *gbu1*Δ::*dpl200 gbu1*Δ::*dpl200 RPS1 rps1*Δ::CIp10-*GBU1*	This work
*agt1*Δ+*AGT1* SBC205	As CAI4 but *agt1*Δ::*dpl200 agt1*Δ::*dpl200 RPS1 rps1*Δ::CIp10-*AGT1*	This work
*car1*Δ+*CAR1* SBC206	As CAI4 but *car1*Δ::*dpl200 car1*Δ::*dpl200 RPS1 rps1*Δ::CIp10-*CAR1*	This work
*gbu1*Δ/*agt1*Δ+*GBU1*/*AGT1* SBC207	As CAI4 but *gbu1*Δ::*dpl200 gbu1*Δ::*dpl200 agt1*Δ::*dpl200 agt1*Δ::*dpl200 RPS1 rps1*Δ::CIp10-*GBU1*-*AGT1*	This work
*gbu1*Δ/*agt1*Δ/*car1*Δ+*GBU1*/*AGT1*/*CAR1* SBC208	As CAI4 but *gbu1*Δ::*dpl200 gbu1*Δ::*dpl200 agt1*Δ::*dpl200 agt1*Δ::*dpl200 car1*Δ::*dpl200 car1*Δ::*dpl200 RPS1 rps1*Δ::CIp10-*GBU1*-*AGT1*-*CAR1*	This work

a Genes (ORF number from *Candida* Genome Database [CGD], NCBI sequence identifier [ID]): *car1* (orf19.3934; XP_721843.1), *gbu1* (orf19.3418; AFP98, KHC73310.1), and *agt1* (orf19.5862; AFP99, XP_723078.1).

10.1128/mBio.01845-20.5 TABLE S1 Lists of orthologues identified in each species used in this study. Download Table S1, TIF file, 0.4 MB. Copyright © 2020 Schaefer et al. 2020 Schaefer et al. This content is distributed under the terms of the Creative Commons Attribution 4.0 International license.

We first assessed whether orf19.5862 and orf19.3418 are required for the utilization of arginine and agmatine and their derivatives (Fig. 1A) or act in an arginase-independent pathway. Spot assays of mutants generated by the CRISP-Cas9 system (Fig. 1B) and the mini-URA blaster gene disruption system (Fig. S2) were performed on minimal media containing intermediate products of those pathways as the sole nitrogen source (Fig. 1B; see also Fig. S2). Arginase belongs to the family of ureohydrolases, and members of this family are involved in the conversion of arginine, agmatine, and 4-guanidinobutyrate (GB) to ornithine, putrescine, and 4-aminobutyric acid (GABA), respectively. Urea is created as a coproduct of this metabolic pathway (25). We therefore examined the suitability of these metabolites as potential nitrogen sources. Yeast extract-peptone-dextrose (YPD) and minimal medium supplemented with (NH_4_)_2_SO_4_ as the nitrogen source served as positive-growth controls.

10.1128/mBio.01845-20.2 FIG S2 Spot assay of C. albicans ureohydrolase mutants generated by the mini-URA blaster gene disruption system on media containing intermediate products of the pathway as a sole nitrogen source. Single, double, and triple mutants of C. albicans orf19.3934 (*CAR1*), orf19.5862 (*AGT1*), and orf19.3418 (*GBU1*) were grown on a range of nitrogen sources relevant to the arginine utilization pathway. Red squares highlight reduced growth of mutant strains, confirming the mutant phenotype generated by the CRISP-Cas9 system shown in Fig. 1B. Inocula with 10-fold dilutions of cultures were spotted on agar containing various nitrogen supplements, and plates were incubated at 30°C and scanned after 72 h. Download FIG S2, TIF file, 0.3 MB. Copyright © 2020 Schaefer et al. 2020 Schaefer et al. This content is distributed under the terms of the Creative Commons Attribution 4.0 International license.

C. albicans wild-type strains were grown on nitrogen-free minimal medium supplemented with (NH_4_)_2_SO_4_, ornithine, urea, arginine, agmatine, 4-guanidinobutyrate (GB), 4-aminobutyric acid (GABA), and putrescine and were shown to be able to utilize all of these as a nitrogen source. All mutant strains were able to grow equally well on ornithine, urea, and GABA (Fig. 1B; see also Fig. S2).

We next demonstrated that C. albicans mutants lacking *CAR1* (*car1*Δ, *gbu1*Δ *car1*Δ, *agt1*Δ *car1*Δ, and *gbu1*Δ *agt1*Δ *car1*Δ) were all affected in growth on minimal medium containing arginine as the sole nitrogen source. These mutants, however, were not affected with respect to their ability to grow on medium containing urea or ornithine, the two products of the arginase reaction. None of the other mutants were affected in growth on arginine, with the single and double mutants of *GBU1* and *AGT1* (orf19.3418 and orf19.5862) demonstrating clear growth (Fig. 1B). Therefore, we conclude that *CAR1* alone encodes arginase activity in C. albicans.

The *gbu1*Δ-1 and *gbu1*Δ-2 single mutants represent two independent clones for the knockout of orf19.3418. Both mutants demonstrated impaired growth on media containing GB, but not GABA, as the nitrogen source, whereas mutants with mutations in *AGT1* (orf19.5862) or *CAR1* exhibited wild-type growth on both (Fig. 1B). Furthermore, the double and triple mutants that lacked orf19.3418 (*GBU1*) also displayed a growth defect on GB but not GABA. This suggests that orf19.3418 (*GBU1*) is likely to encode a GBase, required for the conversion of GB to GABA.

Single mutants with mutations in orf19.5862 (*AGT1*) could utilize all nitrogen sources tested; however, the double mutant lacking both orf19.5862 and orf19.3418 (*gbu1*Δ a*gt1*Δ) and the triple mutant (*gbu1*Δ *agt1*Δ *car1*Δ) were unable to grow on media containing agmatine as the sole nitrogen source (Fig. 1B). Agmatine can act as an immediate substrate for biosynthesis of putrescine, the diamine precursor of spermidine and spermine. As a consequence, in higher eukaryotes that express arginine decarboxylase (26), which converts arginine to agmatine, it participates in an alternative arginase-independent pathway for arginine catabolism and putrescine biosynthesis (Fig. 1A). Alternatively, agmatine may be converted to GB which is then further hydrolyzed to GABA by the enzyme 4-guanidinobutyrase (GBase), which we have identified here as being encoded by *GBU1* (orf19.3418) and which has recently been reported in A. niger (25) and Kluyveromyces lactis (27). The finding that C. albicans mutants lacking both *GBU1* and *AGT1*, but not the corresponding single mutants, failed to grow on agmatine is suggestive of parallel pathways for its utilization also existing in C. albicans. The first pathway would involve the action of *GBU1*, which we have shown to be required for the utilization of GB. The other pathway would involve the conversion of agmatine to putrescine by agmatinase, and our finding that the mutants lacking both *GBU1* and *AGT1* can utilize putrescine as a nitrogen source is therefore consistent with *AGT1* (orf19.5862) encoding an agmatinase. In addition, these findings may exclude the possibility of there being an additional alternative route for putrescene biosynthesis, for example, the agmatine deiminase system that converts agmatine into putrescine via *N*-carbamoyl putrescine in a number of bacterial species (28–31).

These growth assays therefore suggest that *AGT1* and *GBU1* do not encode arginases but that *CAR1* does. Car1 is therefore the only bona fide arginase in C. albicans; this fungus has only one main catabolic pathway for arginine utilization, and this does not appear to have a direct role in agmatine or GB catabolism. C. albicans, however, can utilize agmatine and GB in an arginase-independent manner, and the utilization of agmatine can occur either through its catabolism to GABA by GBase or its catabolism to putrescine by an agmatinase.

### Reduced arginase activity in *car1*Δ mutants does not affect arginase activity or NO synthesis in macrophages.

**FIG 2:**
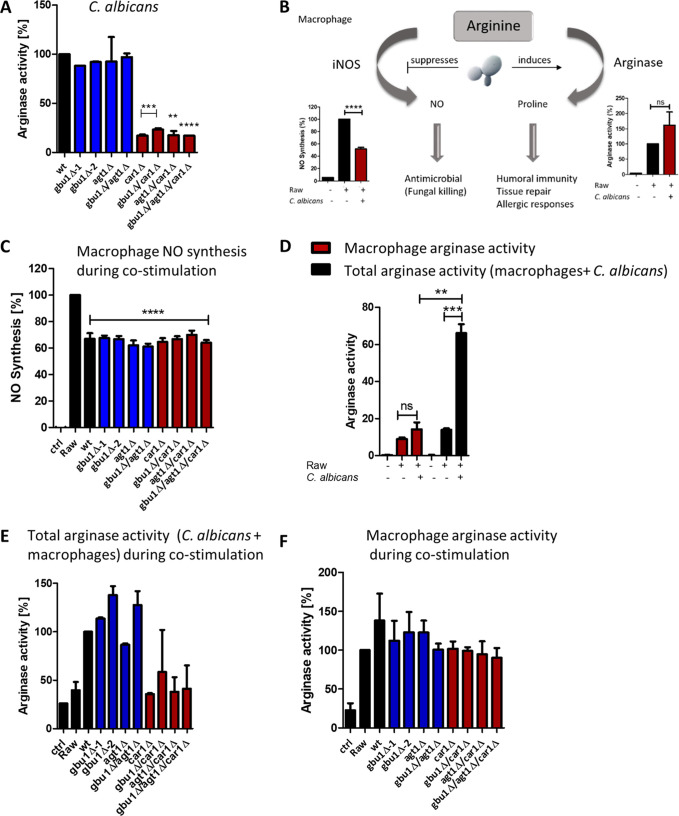
Arginase activity in C. albicans ureohydrolase mutants plus arginase and iNOS activity during the macrophage interaction. (A) Arginase activity was determined in protein extracts isolated from C. albicans cultures grown in YPD. (B) Schematic model of how C. albicans might compete for arginine in competition with macrophages, leading to suppression of NO synthesis by inducing arginase activity (based on data from reference 32). (C) Macrophage NO synthesis following costimulation with C. albicans wild-type and ureohydrolase mutant strains. (D) Macrophage-derived or total (macrophage and C. albicans) arginase activity following costimulation with C. albicans wild-type cells. (E and F) Total (E) or macrophage-derived (F) arginase activity following costimulation with C. albicans wild-type and ureohydrolase mutant strains. **, *P* < 0.01; ***, *P* < 0.001; ****, *P* < 0.0001; ns, not statistically significant.

Consistent with the phenotype seen following growth on arginine as a nitrogen source, the *car1* knockout mutants showed significantly reduced arginase activity following growth in rich media (Fig. 2A, red bars), whereas none of the *agt1* or *gbu1* (single or double) mutants showed any change in arginase activity (Fig. 2A, blue bars). In our previous work, we showed that C. albicans promotes its survival in phagocytes by manipulating the availability of l-arginine (Fig. 2B), which is required for nitrite oxide synthesis by the host (32). All three genes (*CAR1*, *AGT1*, and *GBU1*) were previously shown to be induced strongly in macrophages (33). We therefore wanted to test whether C. albicans Car1, Agt1, and Gbu1 are involved in depleting the macrophage l-arginine pool, thereby reducing the substrate for host inducible nitrite oxide synthase (iNOS) and limiting the synthesis of the antimicrobial nitrite oxide. When macrophages were stimulated with wild-type C. albicans, nitrite oxide production was reduced; however, no statistically significant differences in NO reduction were observed in coculturing performed with any of the gene knockout mutants (Fig. 2C). We next tested if the C. albicans arginase-like enzymes (Car1, Agt1, and Gbu1) impacted macrophage arginase activity following their engulfment. Using two methods of cell breakage to release arginase, we prepared either macrophage-derived or total (macrophage and C. albicans) protein extracts. Increased macrophage arginase activity was seen under conditions of stimulation with wild-type C. albicans cells (Fig. 2D, red bars), as described previously (32), and the total level of arginase activity detected was 3-fold higher (Fig. 2D, black bars) than that seen with macrophages alone. Consistent with Car1 representing the sole arginase activity in C. albicans, this increase in total arginase activity was not seen following costimulation with any of the mutants lacking *CAR1* (Fig. 2E). There were no other statistically significant differences in total arginase activity following costimulation with the other mutant strains. Furthermore, no statistically significant differences were seen in macrophage-derived arginase activity following costimulation with the C. albicans strains (Fig. 2F). Therefore, C. albicans Car1, Agt1, and Gbu1 do not play a direct role in limiting host NO synthesis through substrate limitation and were also not responsible for influencing macrophage arginase or iNOS activity. Other factors produced by C. albicans must therefore lead to the increase in macrophage arginase activity.

### *Candida* arginase Car1 is important for arginine-induced hypha formation.

**FIG 3:**
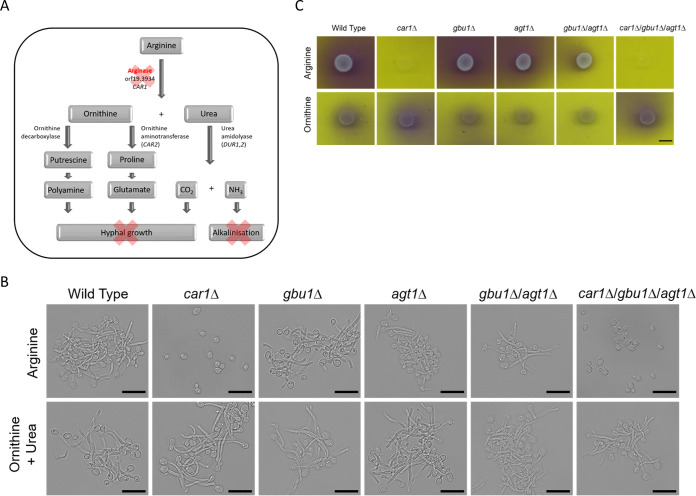
*Candida* arginase *CAR1* is required for hyphal growth and alkalinization. (A) Relationship between arginase activity and subsequent hyphal development and alkalinization as described in the text. (B) Stationary-phase yeast cells were inoculated into induction media containing 0.17% YNB (minus ammonium sulfate), 0.004% glucose, and a 10 mM concentration of either arginine or ornithine and urea. The microscope images shown are representative of results seen after 150 min of incubation at 37°C. The scale bar is 10 μm. (C) C. albicans ureohydrolase mutant spot assay performed on media containing the pH indicator bromocresol purple and either arginine or ornithine as a sole nitrogen and carbon source. Images were taken after 4 days of incubation at 30°C. The scale bar is 5 mm.

Arginine had previously been shown to be important for inducing hypha formation in C. albicans through the cAMP-dependent protein kinase A (PKA) pathway (34). A mutation of the urea amidolyase gene (*DUR1*,*2*), which functions downstream of arginase and hydrolyses urea to NH_3_ and CO_2_, has been found to be defective in arginine-induced hypha formation (34). The CO_2_ produced by this enzyme acts as a signal to stimulate the yeast-to-hypha transition both *in vivo* and in macrophages (34, 35). Furthermore, the resulting alkalinization, through the release of NH_3_, results in a transition from arginine synthesis to arginine catabolism, including increased *CAR1* expression (36) (Fig. 3A). In addition, recent work showed that mitochondrion-generated ATP, in accordance with the conversion of ornithine to proline and its breakdown to glutamate, also plays a key role in arginine-induced morphogenesis and induces hyphal growth more rapidly than the Dur1,2-generated CO_2_ (37) (Fig. 3A). Finally, the breakdown of arginine can also fuel polyamine production, which may also drive morphogenesis through the cAMP-PKA pathway. We therefore tested whether C. albicans Car1, Gbu1, and Agt1 are also involved in arginine-induced hyphal development and alkalinization (Fig. 3). To test for alkalinization, we performed a spot assay on minimal media containing intermediate products of the pathways (Fig. 1A and B) and the pH indicator bromocresol purple (Fig. 3C) to visualize a pH shift from below pH 5.2 (yellow) to above pH 6.8 (purple). We observed alkalization (purple), on medium where arginine was the only nitrogen and carbon source, for the wild-type and *gbu1*Δ and *agt1*Δ single and *gbu1*Δ *agt1*Δ double mutants (Fig. 3C, top images). No color change was observed with the *car1*Δ single mutant and the *gbu1*Δ *agt1*Δ *car1*Δ triple mutant. Alkalinization was abolished when ornithine was added to the medium as a nitrogen and carbon source (Fig. 3C, bottom images). Therefore, the utilization of arginine but not of agmatine or its derivatives leads to alkalinization.

For hypha formation studies, microscopy images were taken after 150 min of induction at 37°C with either arginine or ornithine plus urea (Fig. 3B) or agmatine, GABA, or GB (Fig. S3). Hypha formation was apparent for the wild-type strain and the *gbu1*Δ and *agt1*Δ single and *gbu1*Δ *agt1*Δ double mutants following arginine induction (Fig. 3B, top images). In contrast limited, hyphal growth was observed in the *car1*Δ single and *gbu1*Δ *agt1*Δ *car1*Δ triple mutants, with the majority of the cells remaining in the yeast morphology. Following ornithine and urea induction, all strains, including the *car1*Δ single mutant and *gbu1*Δ *agt1*Δ *car1*Δ triple mutant, developed hyphae (Fig. 3B, bottom images). GB and GABA were also strong inducers of hyphal development, and all C. albicans mutants developed hyphae in their presence. In contrast, agmatine appeared to be a weak inducer of hyphal development under the conditions tested, and again, all of the mutants displayed similar responses (Fig. S3). We therefore conclude that Car1 arginase activity is directly required for arginine-induced hyphal growth and that Car1, alongside Dur1,2, is involved in alkalinization of the environment following growth on arginine.

10.1128/mBio.01845-20.3 FIG S3 C. albicans hyphal growth following induction in 0.17% YNB (minus ammonium sulfate), 0.004% glucose, and a 10 mM concentration of agmatine or GB or GABA. Representative microscope images taken after 150 min of incubation at 37°C are shown. The scale bar is 10 μm. Download FIG S3, TIF file, 0.5 MB. Copyright © 2020 Schaefer et al. 2020 Schaefer et al. This content is distributed under the terms of the Creative Commons Attribution 4.0 International license.

### C. albicans ureohydrolases are required for full virulence.

**FIG 4:**
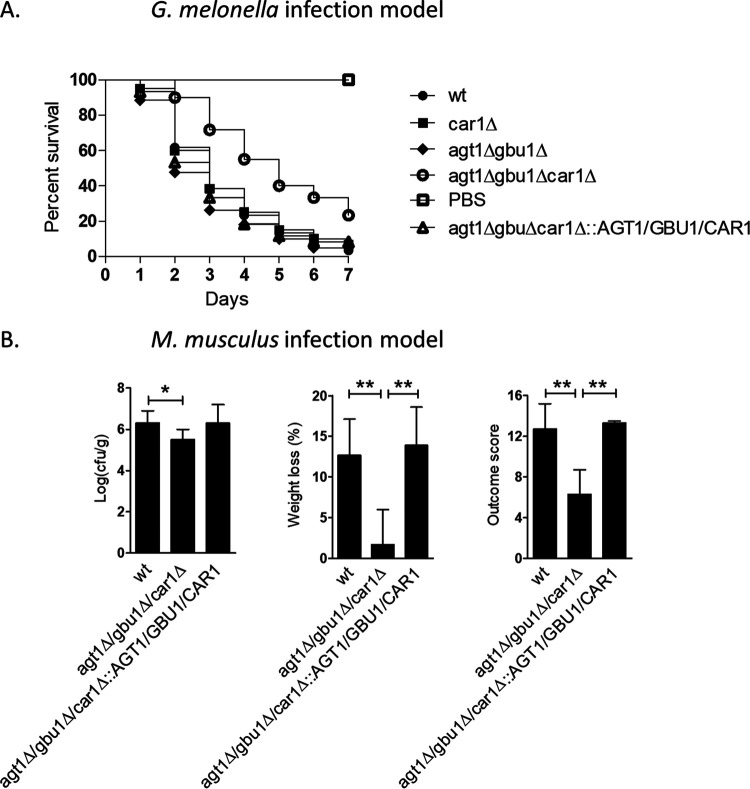
C. albicans arginases family members are required for full virulence in a Galleria mellonella infection model and in a mouse model (Mus musculus) of systemic infection. (A) Survival rate of G. mellonella larvae infected with 8 × 10^4^ cells of C. albicans. Virulence of the wild-type strain and *car1*Δ single, *gbu1*Δ *agt1*Δ double, and *gbu1*Δ *agt1*Δ *car1*Δ triple null mutant strain and of the associated reintegrant control strain was followed for 7 days in a *Galleria* infection model. Reduced virulence was documented for the *gbu1*Δ *agt1*Δ *car1*Δ triple mutant. (B) Virulence of the wild-type, triple null mutant, and reintegrant strains in a 3-day mouse model of systemic infection. Mice were infected with 7.4 × 10^4^ to 7.9 × 10^4^ CFU/g through lateral tail vein infection and sacrificed after 3 days, and the kidney burden (log_10_ CFU/g), percent weight loss, and outcome score (based on both kidney burden and weight loss) were calculated. (**, *P* < 0.01; *, *P* < 0.05).

We next tested whether C. albicans *AGT1*, *GBU1*, and *CAR1* are required for virulence, in both the Galleria mellonella and Mus musculus infection models (Fig. 4). For G. mellonella, larvae were infected with 8 × 10^4^ cells of C. albicans and the survival rate was monitored over 7 days postinfection (Fig. 4A). The virulence of the *car1*Δ single mutant or *gbu1*Δ *agt1*Δ double mutant was indistinguishable from that of the wild type (Fig. 4A). However, the *gbu1*Δ *agt1*Δ *car1*Δ triple mutant displayed significantly reduced virulence and this was rescued in the reintegrant control strain.

A range of mutants was also tested in a 3-day mouse model of systemic infection (Fig. 4B; see also Fig. S4). For this test, CFU counts per gram of kidney tissue and weight loss of the animal were recorded as indicators of disease progression, and the results were combined to calculate a total outcome score (38). No major defect was apparent for the *car1*Δ single mutant or *gbu1*Δ *agt1*Δ double mutant (Fig. S4). However, a significant reduction in fungal burden (*P* < 0.05) was detected for the triple mutant (*gbu1*Δ *agt1*Δ *car1*Δ) and the animals demonstrated significantly reduced weight loss relative to controls (*P* < 0.01), resulting overall in a significantly reduced outcome score (*P* < 0.01). We have therefore demonstrated that *AGT1*, *GBU1*, and *CAR1* together contributed to the virulence of C. albicans in both wax moth and mouse infection models.

10.1128/mBio.01845-20.4 FIG S4 A set of C. albicans of ureohydrolases is required for virulence in a mouse model of systemic infection. Data represent levels of virulence of the wild-type strain or *car1*Δ single, *gbu1*Δ *agt1*Δ double, and *gbu1*Δ *agt1*Δ *car1*Δ triple mutant and reintegrant strains in a mouse 3 day model of systemic infection. Mice were infected with 3.2 × 10^4^ to 3.4 × 10^4^ CFU/g of the *gbu1*Δ *agt1*Δ double mutant strain and associated control strains and with 7.4 × 10^4^ to 7.9 × 10^4^ CFU/g of the *car1*Δ single and *gbu1*Δ *agt1*Δ *car1*Δ triple mutant strains and associated controls through lateral tail vein infection. Mice were then sacrificed after three days, and kidney burden (log_10_ CFU/ml), weight loss (%), and outcome score (based on both kidney burden and weight loss) were calculated (**, *P* < 0.01; *, *P* < 0.05). Download FIG S4, TIF file, 0.1 MB. Copyright © 2020 Schaefer et al. 2020 Schaefer et al. This content is distributed under the terms of the Creative Commons Attribution 4.0 International license.

## DISCUSSION

The objective of this study was to understand the role of arginine metabolism in the *Candida-*host interaction. Previously, we had shown that macrophages that engulf C. albicans, or fungal cell wall chitin, induce host arginase activity (32). This activation could in turn result in protection of the phagocytosed fungus from iNOS, which also uses arginine as a substrate to generate nitric oxide. In addition to the host arginase genes, there are also three genes with high similarity to those encoding arginases in the C. albicans genome (*CAR1*, *AGT1*, and *GBU1*). However, these also display sequence similarity to other arginase-like encoding genes, including agmatinase genes; as such, it was necessary to establish what these gene products were and what role they have in C. albicans development and host interaction. We found that none of these enzymes had an impact on host NO production, either directly via substrate depletion or indirectly through host arginase induction or host iNOS repression. Through nitrogen assimilation profiling of mutants with mutations in these genes, we found that only *CAR1* encodes a bona fide arginase in C. albicans. We propose here that the other two open reading frames, orf19.5862 and orf19.3418, encode agmatinase (Agt1) and guanidinobutyrase (Gbu1), respectively, based on the ability of our set of mutants to utilize specific nitrogen substrates. While arginase metabolism and arginine metabolism have been studied in a range of fungal species (39), relatively little is known about the role of agmatinase or guanidinobutyrase in fungi.

Recently, agmatinase was characterized in the filamentous fungus Neurospora crassa (*AGM-1*) and it was shown to have an impact on F-actin dynamics and to play an essential role in cell development (40). Interestingly, N. crassa Δ*agm-1* null mutants were unable to grow and the heterokaryon strain showed reduced hypha elongation, conidiation, and biomass production despite retaining ornithine decarboxylase activity (40). Arginine decarboxylase, the enzyme that produces the agmatine for agmatinase, whose decarboxylation yields putrescine, has previously been described in bacteria (41, 42), animals (43), and plants (44). Our searches revealed no arginine decarboxylase homologue in C. albicans, confirming that only four fungi have been identified to have arginine decarboxylase to date (7–9). It may be that the absence of arginine decarboxylase is correlated with the occurrence of ornithine decarboxylase and indicates that fungi normally synthesize putrescine directly by decarboxylation of ornithine. Plants and archaebacteria may also form putrescine from agmatine via agmatine deiminase and *N*-carbamyl-putrescine hydrolase (45, 46). However, no homologous gene or evidence for this activity has been found in C. albicans. In K. lactis, an alternative arginase-independent pathway for arginine metabolism has been described that may be widespread in fungi (27). This pathway involves the transamination of arginine to ketoarginine, which is then decaboxylated to guanidinobutanal and oxidized to 4-guanidinobutyrate (4-guanidinobutyric acid or GB). This pathway for arginine catabolism would operate as an alternative to the arginine decarboxylase that is absent in fungi and would also provide a potential alternative to ornithine decarboxylase. Deletion of the arginase gene *CAR1* in C. albicans did not completely abolish growth on arginine as a sole nitrogen source, as background growth of the *car1*Δ mutant was maintained on this medium. This may suggest that C. albicans may also have an alternative pathway for arginine catabolism, potentially through ketoarginine activity as described in K. lactis.

We show that GB could be used as the sole nitrogen source by C. albicans, as has also been described for Penicillium roqueforti (47) A. niger (25), and K. lactis but not S. cerevisiae (27). Indeed, GBase, the enzyme that catalyzes the hydrolysis of GB to GABA and urea, has been shown to be widespread in fungi (27), although it is absent in some *Saccharomycetaceae* clades, including the majority of S. cerevisiae strains sequenced (27). In K. lactis, the *KlGBU1* gene was originally described as encoding an arginase-like enzyme before its true biochemical activity was revealed. It is the homolog of orf19.3418 (*GBU1*) in C. albicans, which we describe here as acting as a GBase gene.

We show that C. albicans can utilize agmatine as a sole nitrogen source via two independent pathways (Fig. 1A). First, agmatine catabolism can occur via the guanidinobutyrase (Gbu1) pathway that produces GABA and succinate. Alternatively, agmatinase may act to produce putrescine from agmatine. The action of an agmatinase would represent an alternative to the arginase and ornithine decarboxylase pathway that ultimately yields polyamines or GABA. These arginase-independent pathways link agmatine to succinate, either through putrescine by agmatinase activity or guanidinobutyrase by GBase activity. It is not known whether C. albicans can synthesize agmatine, but we show that this fungus is capable of assimilating externally provided agmatine via agmatinase or guanidinobutyrase (GBase; *GBU1*). Although the physiological significance of these pathways is not clear, they may provide potential growth advantages under conditions in which C. albicans encounters agmatine as a source of carbon and nitrogen. In fungi that have all three enzymes, agmatinase and guanidinobutyrase are predicted to be secreted enzymes whereas arginase is cytoplasmic. It may be that Car1 is required for central intracellular metabolism, whereas the others may function to scavenge carbon and nitrogen from the environment by breaking down complex nutrients such as agmatine. Alternatively, uptake of agmatine and GB may be limited, and their utilization would consequently be dependent on initial extracellular degradation.

Agmatine has been detected in many organs in rats, but the stomach (71 ng g^−1^ wet weight), small intestine (55 ng g^−1^), and large intestine (28 ng g^−1^) have the highest agmatine tissue concentrations (48, 49). The agmatine present in the lumen of the human gastrointestinal tract is derived from three sources (19). Agmatine is produced and released by gut bacteria of the human microbiome (18, 50, 51) or from ingested food (19), or luminal agmatine derives from desquamated intestinal epithelial cells (18). Therefore, the ability of C. albicans to utilize agmatine may provide a fitness benefit for the fungus in the human host. In S. cerevisiae fermentation, it is known that arginine catabolism is also involved in the production of proline, which cannot be assimilated under anaerobic conditions (52). Arginine catabolism occurring through the guanidinobutyrase pathway could therefore provide a selective advantage under anaerobic and nitrogen-limited conditions.

Polyamines (putrescine and spermidine) are important metabolites of intestinal bacteria in humans, and the concentrations of putrescine in the intestinal lumen of a healthy individual range from 0.5 to 1 mM (53, 54). Food-derived polyamines are normally absorbed before they reach the lower intestine (55); therefore, most polyamines in the large intestine are derived from the intestinal microbiota (56). A novel pathway for putrescine production from arginine through agmatine has been reported that involves the collaboration of two bacterial species and is triggered by environmental acidification (57). In this pathway, arginine can be taken up from the environment by Escherichia coli (arginine-dependent acid resistance system) and converted by arginine decarboxylase to agmatine, which is then exported to the medium (57). Bacteria that have the agmatine deiminase system, such as Enterococcus faecalis, can then take up the released agmatine and convert it to putrescine. *In silico* metagenome analysis of the most abundant bacterial species in the human microbiome (58) has suggested that many species are unable to produce putrescine from arginine (59, 60), indicating that agmatine metabolism may involve a consortium of cross-feeding bacteria. It may be that similar interspecies metabolic communication occurs between C. albicans and bacteria of the intestinal microbiota, where the fungus is able to assimilate bacterially produced agmatine.

In addition, agmatine can serve as an endogenous human cell-signaling molecule that triggers an innate immune response, which can enhance the immune response during infection (28, 61). It might be that organisms that express agmatine-degrading enzymes possess a mechanism that potentially evades this component of the human immune system. A similar role for Helicobacter pylori arginase has been proposed where the depletion of arginine attenuates the innate immune response (62). It is therefore possible that C. albicans utilization of agmatine may indirectly dampen the host immune response.

### Conclusion.

The results presented suggest a novel route for polyamine metabolism and agmatine assimilation in the fungal pathogen C. albicans. We show that agmatine can be assimilated by C. albicans using two independent ureohydrolase-dependent pathways that use agmatinase or guanidinobutyrase. The ureohydrolase family that we posit, comprising arginase (Car1), agmatinase (Agt1), and guanidinobutyrase (Gbu1), represents an enzyme set whose members are collectively essential for C. albicans virulence. This set of ureohydrolases does not affect macrophage arginase or iNOS activity and also does not function in protection from the phagocytic burst but enables arginine and arginine-like substrates to support growth during pathogenesis.

## MATERIALS AND METHODS

### Strains and culture condition.

C. albicans strains used in this work are listed in Table 1 and were maintained as glycerol stocks at −70°C. Cells were grown in YPD medium (1% [wt/vol] yeast extract, 2% [wt/vol] peptone, 2% dextrose) at 30°C and 200 rpm. When solid medium was required, 2% (wt/vol) agar was added. Cells from overnight cultures (YPD at 30°C, 200 rpm) were used to inoculate fresh YPD broth until the exponential-growth phase was reached. Cells were collected, washed twice with phosphate-buffered saline (PBS), counted, and used for costimulation of macrophages. To generate mutants using the CRISPR-Cas9 strategy, transformants were selected on YPD agar supplemented with 200 μg/ml nourseothricin (ClonNAT; Werner BioAgents, Jena, Germany). For generation of the mutant strains by the use of a *URA3* recyclable PCR-mediated gene disruption system (23), transformants were selected on SC-U medium (0.67% yeast nitrogen base [YNB], 2% glucose, 0.077% complete supplement mixture minus uracil). The *URA3* marker was then recycled through selection on SC-U medium plus 5-fluoroorotic acid (2 mg/ml) and uridine (50 μg/ml). For spot assay, cells were grown in minimal medium (2% glucose, 0.17% YNB [without amino acids and ammonium sulfate], 10 mM NH_4_SO_4_) washed twice with H_2_O, and spotted on minimal agar media (2% agar [without NH_4_SO_4_]) containing 10 mM concentrations of the indicated nitrogen sources. Solutions of urea (Sigma, CAS 57-13-6), ornithine (Sigma, CAS 3184-13-2), arginine (Sigma, CAS 74–75-3), agmatine (Sigma, CAS 2482-00-0), 4-guanidinobutyrate (GB; Santa Cruz Biotechnology, CAS 463-00-3), 4-aminobutyrate (GABA; Sigma, CAS 56-12-2), and putrescine (Sigma, CAS 333-93-7) were subjected to sterile filtration and added to the media after autoclaving. To monitor alkalinization, the pH indicator bromocresol purple (Sigma, CAS 115-40-2) was added (0.01%) to medium with or without glucose as a carbon source. YPD and minimal medium containing 10 mM NH_4_SO_4_ served as the control. Prior to virulence testing, strains were grown in NGY medium (0.1% neopeptone, 0.4% glucose, 0.1% yeast extract) at 30°C for 16 h.

Cells of the RAW 264.7 (ATCC TIB-71) murine macrophage cell line were cultured in Dulbecco’s modified Eagle’s medium (DMEM) (Sigma), supplemented with 2 mM l-glutamine, 100 U/ml penicillin, 100 mg/ml streptomycin, and 10% (vol/vol) fetal bovine serum, at 37°C and 5% (vol/vol) CO_2._ After reaching 90% confluence, cells were washed with PBS to remove dead cells and detached using 10 mM EDTA and 4 mg/ml lidocaine–HCl–PBS for 5 to 10 min. Cells were harvested (10 min, 300 × *g*) and either reinoculated for further cultivating or subseeded into 12-well plates at a density of 5 × 10^5^ cells/well in the media described above supplemented with 1% minimum essential medium (MEM) and nonessential amino acids (NEAA; Gibco) (100×) and left overnight to adhere. Macrophages were activated with 100 ng/ml gamma interferon (IFN-γ) and lipopolysaccharide (LPS) 4 h prior to coincubation with 2.5 × 10^6^ C. albicans cells (macrophage/yeast cell ratio of 1:5) for 3 h. Supernatant was collected and stored at –20°C for arginase activity and nitric oxide measurements.

### Genetic manipulation.

For gene disruption using the CRISPR-Cas9 strategy, C. albicans strain SC5314 (63) was used as the parental strain. The CaCas9 Solo system (pV1200 vector) was used in this work, and constructions of knockout and reconstitution vectors, marker recycling, and verification of CRISPR-mutagenized loci were performed according to a previously described method (24, 64). Briefly, single guide RNA (sgRNA) sequences were selected (the sequences can be found at http://osf.io/ARDTX) targeting the C. albicans open reading frame of interest directly upstream of a NGG protospacer-adjacent motif (PAM) site. Overhang sequences of annealed and phosphorylated 20-base forward and complement reverse guide oligonucleotides were ligated into CIP (calf intestinal phosphatase-treated BsmBI-digested pV1200 parent vector, and correct clones were identified by sequencing. The vector (5 to 10 μg) was then linearized by digestion with KpnI and SacI before transformation was performed for efficient targeting to the *ENO1* locus. Transformation was performed along with processing using a purified repair template (3 μg) that was generated with 60-bp oligonucleotide primers containing a 20-bp overlap at their 3′ ends centered on the desired mutation point. Homozygous mutations (introduced restriction site, stop codon, a frameshift, and mutation of the corresponding PAM) were verified by sequencing (GENEWIZ; Takeley Sanger Sequencing Laboratory, United Kingdom). Reconstitution of the wild-type genotype from the null mutant background was performed with the CRSIPR-Cas9 transformation protocol described above. Both alleles were restored to the wild-type form (involving removal of the introduced restriction site, the mutated PAM, and the stop codon and correction of the frameshift). However, the reconstituted wild-type strains included the introduction of a silent PAM mutation. The genotypes of the transformants were verified by sequencing. Details of the primer sequences used are listed in Table S2 and Table S3.

10.1128/mBio.01845-20.6 TABLE S2 Oligonucleotide sequences used for gene disruption by the CRISP-Cas9 system in this study. Download Table S2, TIF file, 0.3 MB. Copyright © 2020 Schaefer et al. 2020 Schaefer et al. This content is distributed under the terms of the Creative Commons Attribution 4.0 International license.

10.1128/mBio.01845-20.7 TABLE S3 Oligonucleotide sequences used for gene restoration by the CRISP-Cas9 system in this study. Download Table S3, TIF file, 0.2 MB. Copyright © 2020 Schaefer et al. 2020 Schaefer et al. This content is distributed under the terms of the Creative Commons Attribution 4.0 International license.

Gene disruption was also conducted using the PCR-directed mini-URA blaster method as previously reported (23). Briefly, disruption cassettes were amplified from plasmid pDDB57 using long primers (Table S4) containing 70 bp of sequence with homology to the target ORF. Mutants were made through the sequential transformation of the disruption cassettes and recycling of the *URA3* marker through selection on 5-fluoroorotic acid. Ultimately, to avoid issues associated with the ectopic expression of *URA3*, the *URA3* marker was integrated into the *RPS1* locus of all mutants by transformation with StuI-digested CIp10 plasmid (65). For reintegrant controls, the genes, plus ∼1,000 bp upstream and ∼500 bp downstream, were PCR amplified and cloned into the XbaI, NotI, or HindIII site of CIp10 for *CAR1*, *GBU1*, or *AGT1*, respectively. The required double and triple reintegrant control vectors were made through sequential cloning, and the vectors were ultimately linearized through digestion with StuI and integrated at the *RPS1* locus.

10.1128/mBio.01845-20.8 TABLE S4 Oligonucleotide sequences used for gene disruption by the mini-URA blaster system and construction of reintegrant cassettes. Download Table S4, TIF file, 0.1 MB. Copyright © 2020 Schaefer et al. 2020 Schaefer et al. This content is distributed under the terms of the Creative Commons Attribution 4.0 International license.

### Phenotypic characterization of mutants.

For spot assays, exponentially growing cells were harvested from minimal medium at 30°C, washed twice with deionized water, and adjusted to an optical density (OD) of 0.1. The samples were then serially diluted, and 3 μl was spotted onto the different media. Plates were incubated at 30°C, checked at regular intervals, and scanned after 72 h (except for the YPD and arginine-containing media, where end points were recorded after 48 h and 96 h, respectively). For hyphal induction, strains were grown initially in minimal medium (0.67% YNB, 2% glucose) for 24 h; the cells were then washed and inoculated into prewarmed induction media at 1 × 10^6^ cells/ml and incubated at 37°C for 150 min. The induction media consisted of 0.17% YNB (minus ammonium sulfate) and 0.004% glucose supplemented with either 20 mM arginine, 20 mM urea and ornithine, 20 mM agmatine, 20 mM 4-guanidinobutyrate (GB), or 20 mM 4-aminobutyrate (GABA).

Macrophage and C. albicans cocultures were washed once with ice-cold PBS before macrophages were lysed for 10 min by adding 200 μl of 0.4% Triton X-100–10 mM Tris-HCl (pH 7.4) supplemented with fresh 1 μM pepstatin A and 1 μM leupeptin in each well. Samples were collected, transferred into centrifugation tubes, and centrifuged for 10 min at 4°C and 13,000 rpm to remove cellular debris. Lysates were transferred into fresh tubes and stored at −20°C until analyzed. For *Candida* cell lysis, protein lysis buffer (50 mM Tris, 150 mM NaCl, 10 mM imidazole, and 0.1% NP-40, with freshly added protease inhibitors [1 μM pepstatin A and 1 μM leupeptin]) was added to each well and incubated 5 min on ice. Cells were scraped from the surface and transferred into cold centrifugation tubes, snap-frozen in liquid nitrogen, and stored at –20°C before protein extraction. Pellets were thawed at 4°C, washed, and resuspended in 350 μl ice-cold lysis buffer and transferred to a ribolyser tube containing 1 ml chilled glass beads. Samples were disrupted by bead beating (BioSpec) performed twice for 15 s each time. Protein concentrations were determined using Bradford reagent (Bio-Rad standard assay in microplates). Arginase activity and nitric oxide concentrations in supernatants and cell lysates were determined using an arginase activity assay kit (Sigma-Aldrich) and a Griess reagent kit for nitrite determination (Molecular Probes) according to the instructions of the manufacturers.

### Statistical analysis.

Multiple-protein-sequence alignment (see Fig. S1A in the supplemental material) was performed using MUSCLE (66), and the results were used to calculate the percentages of identity of the three proteins (Fig. S1B). OMA (Orthologous MAtrix) database v2.3.0 (67) was used to identify orthologues among publicly available (see Table S1 in the supplemental material), complete genome sequences and identified pairwise orthologues between all pairs of species in the analysis (Fig. S1C). To guarantee that a significant fraction of a sequence was aligned, a length tolerance criterion was used. According to the criterion used, the length of the shorter aligned sequence was required to represent at least fraction ℓ of the longest sequence. The relationship was expressed as minimum (|a1|, |a2|) > ℓ·maximum (|s1|, |s2|), where a1 and a2 are the lengths of the aligned subsequences of s1 and s2. Alignments that satisfy both the length and score criteria are upgraded to candidate pairs (CP). OMA was run with default parameters apart from the length tolerance criterion, which was lowered from 0.61 (default) to 0.45 to include the manually identified BLAST results (∼80% identity; E value < 1e−70; bit score > 250) corresponding to a C. auris ortholog that was truncated to ∼0.5 the length of C. albicans CAR1 (194 amino acids compared to 319 for C. albicans CAR1).

Statistical analyses of G. mellonella and M. musculus virulence assay results (Fig. 4) were performed using GraphPad Prism 5 software. All the G. mellonella experiments were performed with three biological replicates in duplicate. Data represent cumulative results of all experiments performed, and errors were calculated as means ± standard deviations (SD). Log rank tests were conducted for the G. mellonella survival assays, and analysis of variance (ANOVA) or Dunnett’s test was used for the M. musculus model to establish statistical significance, which was set at a *P* value of <0.05.

### Virulence assay.

Galleria mellonella larvae were purchased from the United Kingdom (Waxworms Ltd., Sheffield, United Kingdom). Groups of 20 healthy larvae (0.25 to 0.35 g) were inoculated through the last left proleg into the hemocoel with 10 μl of cell suspension (8 × 10^4^ cells/larva) using a Hamilton syringe. Following infection, larvae were incubated in the dark at 37°C and survival was monitored daily for 7 days on the basis of responses to physical stimulation and melanization. Larvae inoculated with PBS were used as uninfected controls, and the experiments performed with the uninfected controls resulted in no deaths. Assays were performed at least 3 times independently (Fig. 4A). Female BALB/c mice were originally purchased from Charles River, United Kingdom, and provided with food and water *ad libitum*. Mice were infected with an infectious dose of 7.4 × 10^4^ to 7.9 × 10^4^ CFU/g through a lateral tail vein and were humanely sacrificed after 3 days, and the kidney burden (log_10_ CFU/ml), percent weight loss, and outcome score (38) (based on both kidney burden and weight loss) were calculated (**, *P* < 0.01; *, *P* < 0.05).
