# Supplementary figures and images for: Three Related Enzymes in Candida albicans Achieve Arginine- and Agmatine-Dependent Metabolism That Is Essential for Growth and Fungal Virulence
Source: mBio. 2020 Aug 11;11(4):e01845-20. doi: 10.1128/mBio.01845-20 (PMC7439472; doi:10.1128/mBio.01845-20)

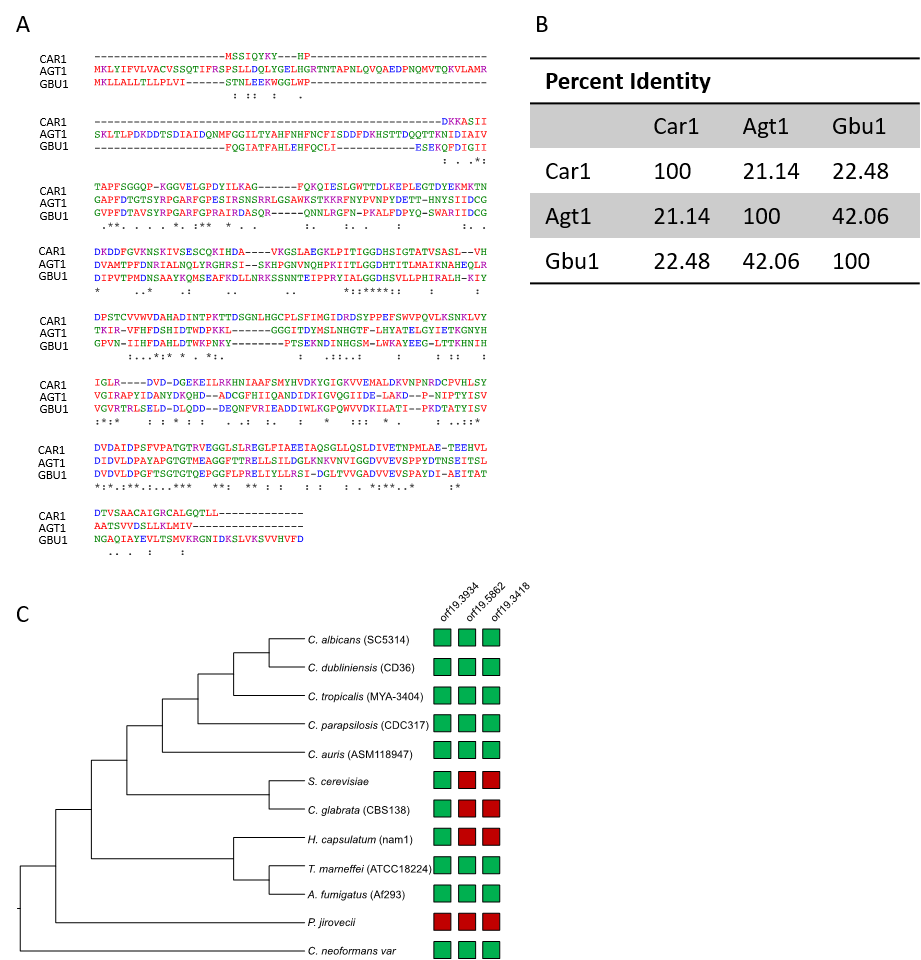

Supplement: FIG S1 [file mBio.01845-20-sf001.tif]

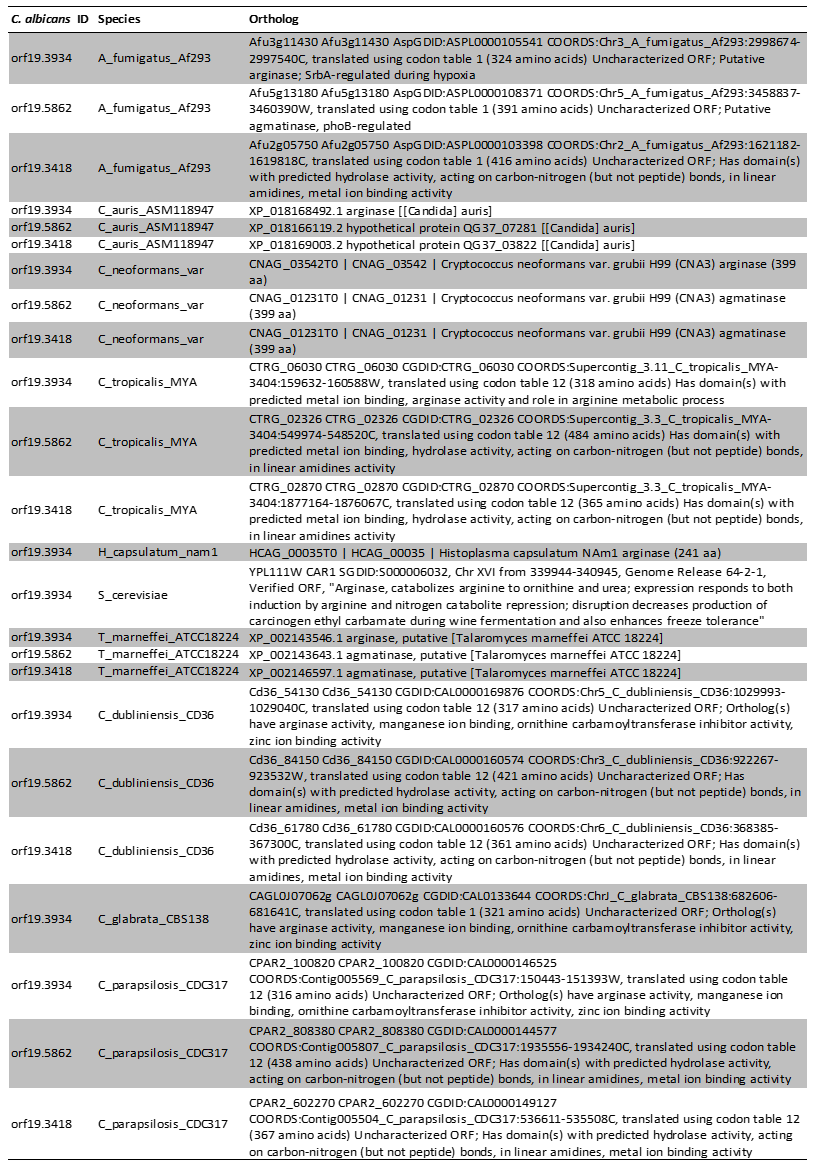

Supplement: TABLE S1 [file mBio.01845-20-st001.tif]

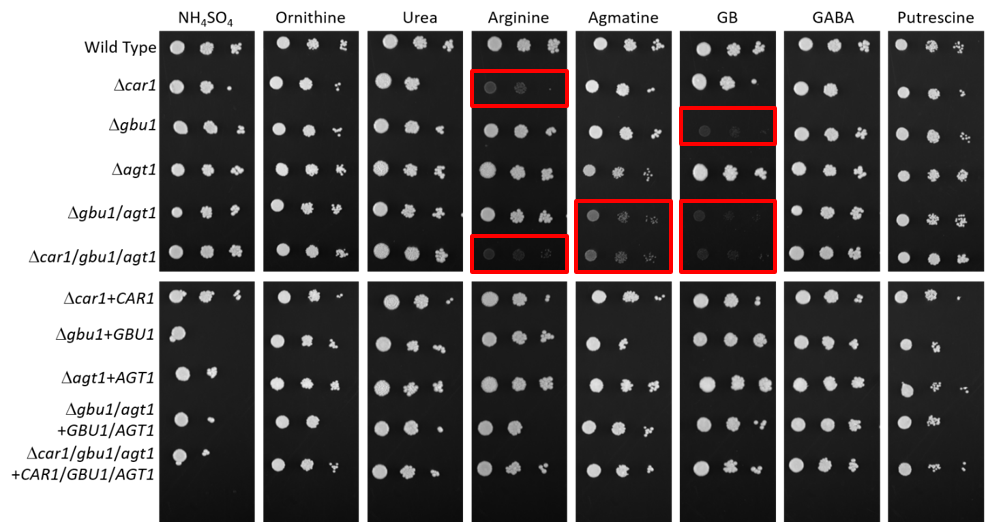

Supplement: FIG S2 [file mBio.01845-20-sf002.tif]

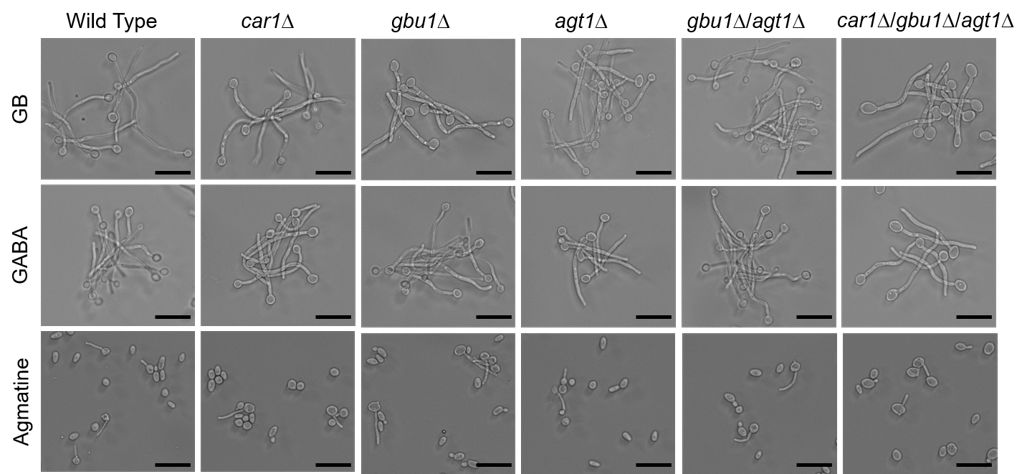

Supplement: FIG S3 [file mBio.01845-20-sf003.tif]

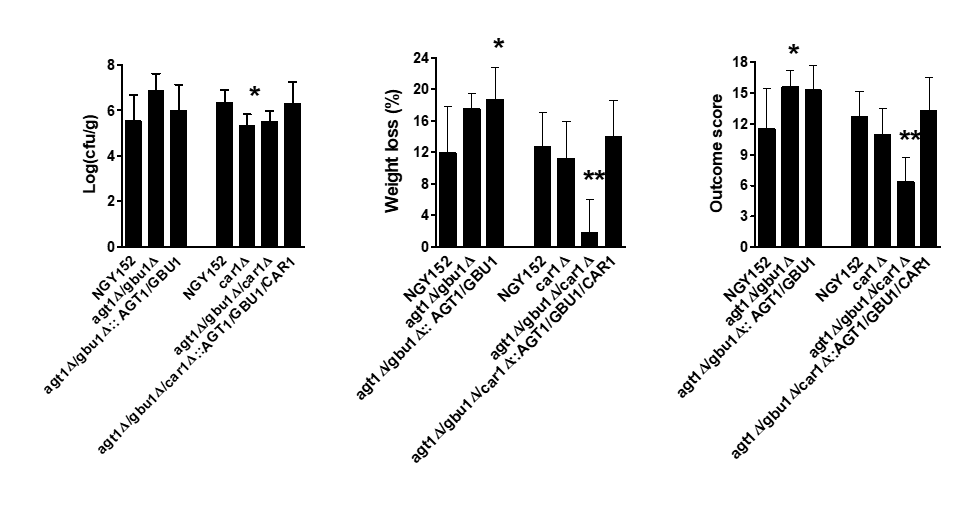

Supplement: FIG S4 [file mBio.01845-20-sf004.tif]

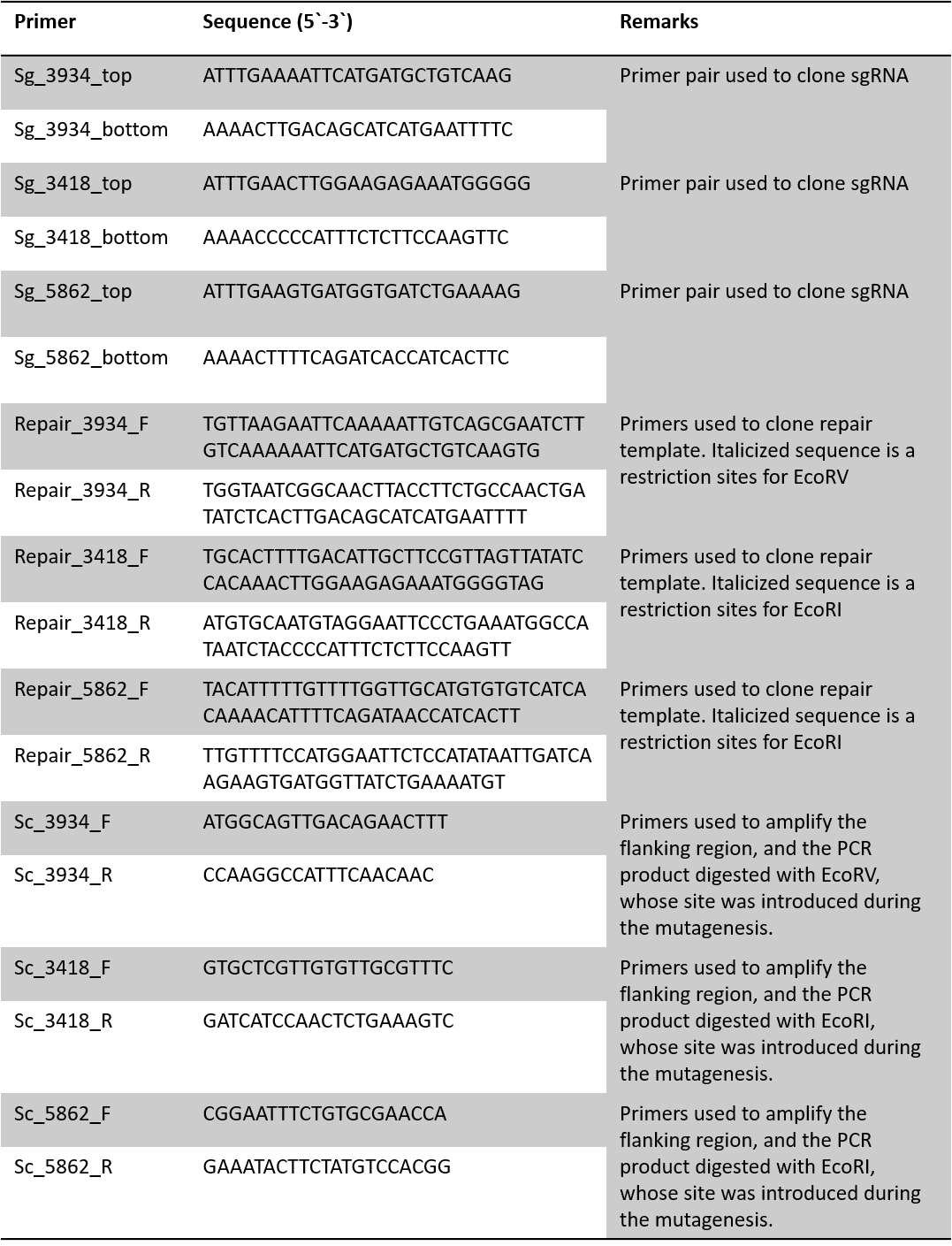

Supplement: TABLE S2 [file mBio.01845-20-st002.tif]

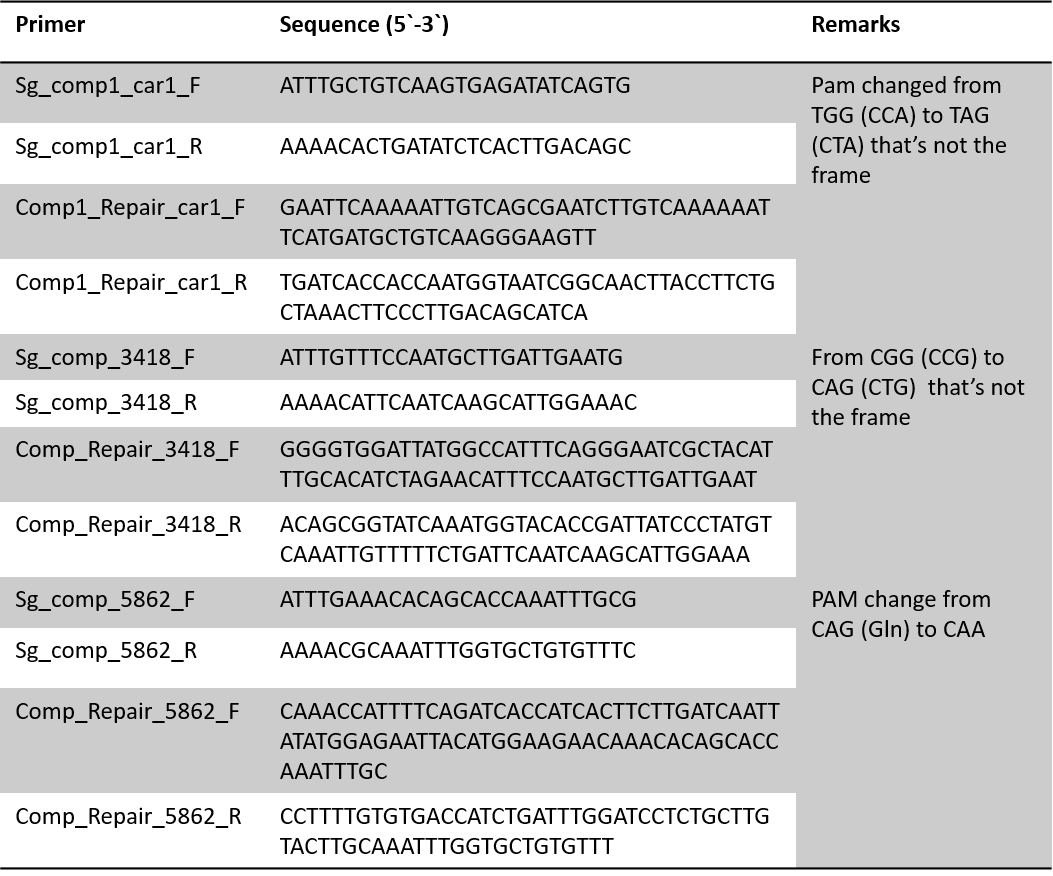

Supplement: TABLE S3 [file mBio.01845-20-st003.tif]

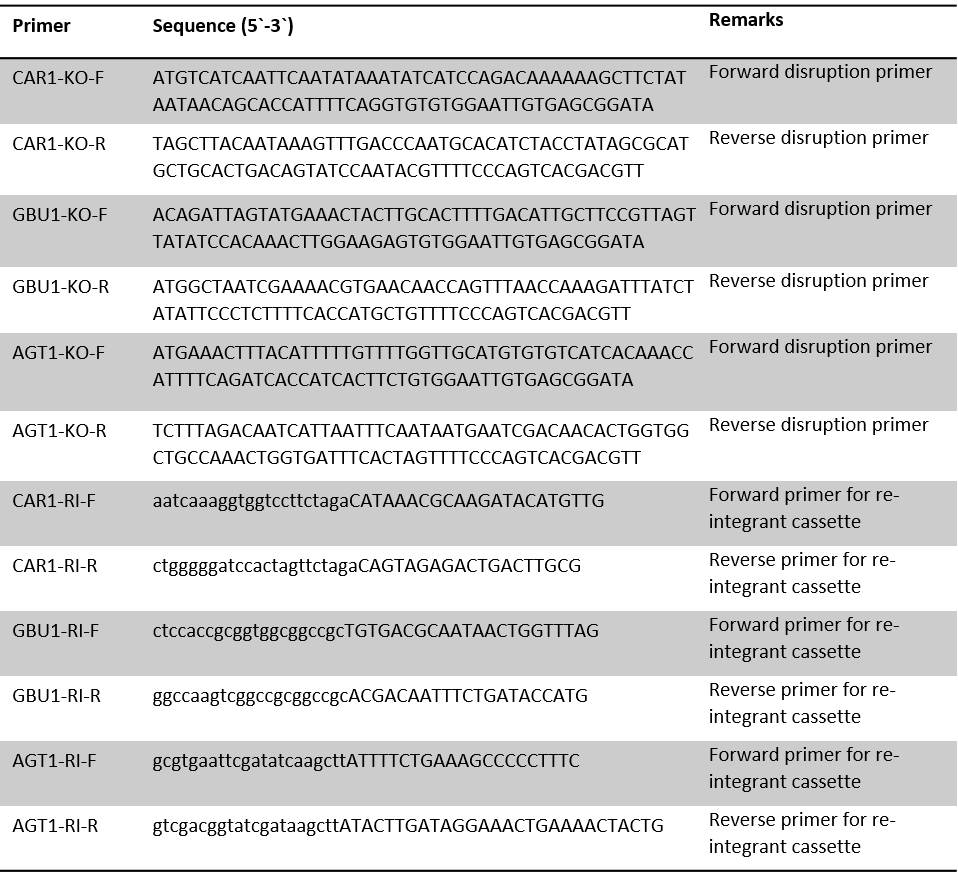

Supplement: TABLE S4 [file mBio.01845-20-st004.tif]
